# Molecular docking enabled updated screening of the matrix protein VP40 from Ebola virus with millions of compounds in the MCULE database for potential inhibitors

**DOI:** 10.6026/97320630015627

**Published:** 2019-10-08

**Authors:** Hasanain Abdulhameed Odhar, Ali Mahmood Rayshan, Salam Waheed Ahjel, Alaa Abduljabbar Hashim, Ali A. Mohammed Ali Albeer

**Affiliations:** 1 Department of pharmacy, Al-Zahrawi University College, Karbala, Iraq

**Keywords:** Ebola virus, VP40, virtual screening, docking, lead-likeness, mcule.com

## Abstract

Ebola virus is known for several outbreaks of hemorrhagic fever in West Africa. This RNA virus is linked to high fatality and easy transmission. Recently, an effective vaccine and a monoclonal antibody cocktail have been introduced to combat Ebola virus infection. The matrix protein VP40 of Ebola virus is a known drug target and it is essential for viral life cycle through participation in RNA transcription as well as for the budding of the mature virus. It is known that residues phenylalanine 125 and arginine 134 of VP40 are involved in the interaction with RNA. Therefore, it is of interest to screen VP40 with millions of compounds at the mcule.com database for potential inhibitors. The output hits were ranked according to their minimum binding energy to matrix protein VP40. We further calculated the pharmacokinetics and toxicology properties for the best five hits using several predictive ADME analysis web tools. We report a candidate lead (compound #5: ((10R)-10-(4-hydroxyphenyl)-11,12,14,16-tetraazatetracyclo[7.7.0.02,7.011,15] hexadeca-1(16), 2(7),3,5,8,12,14-heptaen-8-ol)) with high drug-likeness score, promising lead-likeness behaviour and high median lethal dose. The candidate lead compound #5 engages in hydrogen bonding and hydrophobic interactions with VP40 active site residues. Thus, the lead compound #5 is recommended for further in vitro and in vivo validations for further consideration.

## Background

The Ebola virus (EboV) is an antisense RNA virus that had been responsible for several outbreaks of hemorrhagic fever mainly in West Africa. This virus has been categorized as a bio-weapon hazard due to its high transmission capacity with fatality rate of about 90%[1,2].Attempts to combat Ebola virus infection with an effective vaccine or apotential drug has been crowned with the development of a recombinant vesicular stomatitis virus Ebolavaccine and a monoclonal antibody cocktail named ZMapp [3,4].Additionally, computational approaches are currently employed to generate novel anti-EboV compounds.These approaches are also applied nowadays to repurpose some FDA approved drugs like ibuprofen for treatment and prevention of EboV[5].The RNA genome of Ebola virus is composed of about 19 Kb nucleotides that encodes for seven structural proteins.These conserved proteins include glycoprotein (GP), matrix protein VP40, VP35, VP30, VP24, nucleoprotein (NP) and polymerase L protein.These proteins can serve as potential drug targets for high throughput screening (HTS) [1,6].

**Figure 1:**
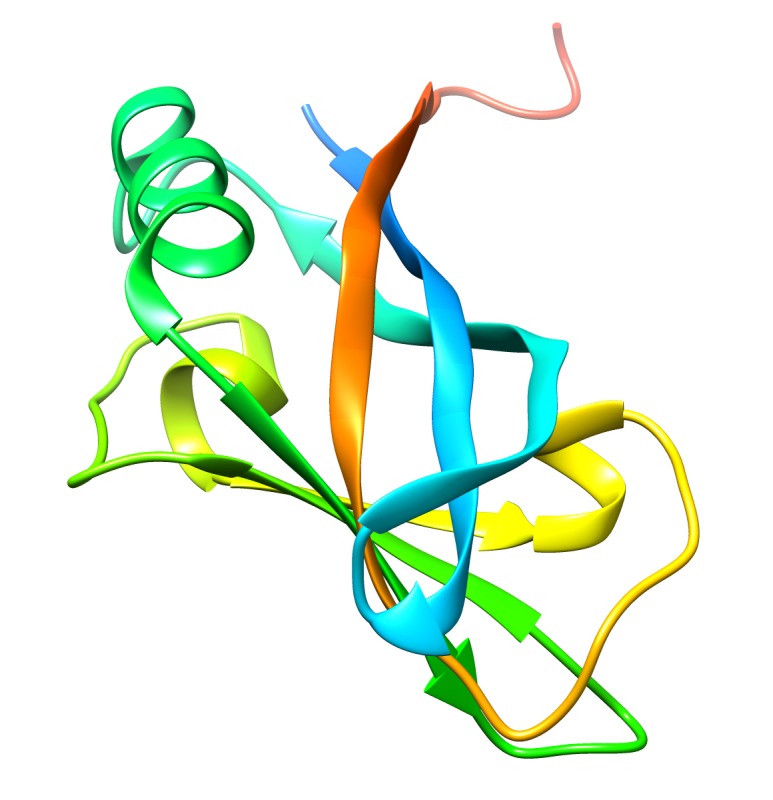
A three-dimensional cartoon representation of EboV matrix protein VP40 crystal. VP40 crystal structure with code 1H2C was retrieved from protein data bank [18] and visualized by using UCSF chimera version 1.13.1[19]. The C-terminus is colored by red while N-terminus is colored with blue.

The matrix protein VP40 is localized below Ebola virus envelope and it is considered the most abundant viral protein[7]. VP40 plays multiple essential roles within EboV life cycle likein maturation of the virus, regulation of RNA transcription and also budding of the mature virus from host plasma protein [8,9]. Furthermore, VP40 may also contribute to the progression of Ebola virus disease pathogenesis through incorporation into exosomes. This incorporation step can intensify EboV pathogenesis by adversely impacting the viability of host immune cells [10,11].The crystallographic images of VP40-RNA complex clearly shows that both Arginine 134 and Phenylalanine 125 residues of VP40 are mainly involved in interactions with RNA [12].These interactions seem to be essential for octamer formation of VP40 and viral transcription in early stages of infection. Mutation studies indicate that both Arginine 134 and Phenylalanine 125 mutations can adversely influenceEboV RNA binding and VP40 octamer formation[13]. These studies suggest that the interactions between EboV RNA and VP40 octamer is critical for viral life cycle and may be a probable target for the design and discovery of new anti-EboV drugs [11].A three-dimensional structure representation of matrix protein VP40 can be seen in Figure 1. It is worth mentioning that the blue colored N-terminal domain as seen in Figure 1 is believed to be responsible for VP40 oligomerization into different conformations.These various conformations may explain the different cellular functions carried out by VP40 within Ebola virus life cycle [12,14].

Several previous studies had virtually screened large chemicals databases like zinc database and traditional Chinese medicine (TCM) database [15,16]. In these in silico projects, the binding potential of these chemicals against VP40 of Ebola virus was assessed and several pharmacokinetics parameters were estimated. In this project, we have virtually screened the full version ofmcule.com chemical database [17] against matrix protein VP40 of EboV. The minimum binding free energy of the top five hits were reported and analyzed. Several chemical, pharmacokinetics and toxicological parameters were also estimated for these top chemicals.Our aim is to identify potential lead compounds for probable use against Ebola virus infection by using computational approach.

## Methodology

### Structure-based virtual screening:

We have used mcule.com [17] online platform to carry out our hits identification workflow. This platform provides a collection of online drug discovery tools and well-curated chemicals databases. In summary, we used MCULE purchasable (full) database as updated on July 2019.This version of database contains more than 42 million chemical compounds. For our screening protocol, we used default options to select our final hits. It is worth to mention that we have implemented both sampler and diversity filters to randomly select diverse and different chemical structures with no more than 10 rotatable bonds, 5 chiral centers and 1 violation of Lipinski's rule of five. These filters can save our online tools limit and speed upour preliminary screening process.In order to eliminate the possibility of havingpromiscuous ligands and minimize non-selective and frequent hitters, we have added a REOS (rapid elimination of swill) filter.Finally, the filtered chemicals were virtually screened for binding potential against Ebola virus matrix protein VP40 by using AutoDockvina. The crystal structure of VP40 with code 1H2C was downloaded from protein data bank [18]. Both water molecules and bounded ligand were removed from VP40 crystal by using UCSF chimera version 1.13.1[19].Then, VP40 crystal was uploaded as PDB file into docking (vina) platform. Upon uploading target protein, AutoDock online tools have automatically processed the target by adding polar hydrogen atoms and Gasteiger charges.We have used a binding site area in Angstrom of (22*22*22) with coordinates of (X:5, Y: 18 and Z: 25).We have also used the maximum allowed online exhaustiveness of 2.The final output compounds were ranked based on their minimum binding energy to VP40 crystal.

### Docking analysis:

To elaborate our understanding of ligand-target interactions, we repeated virtual docking studies for the top five hits generated with mcule.com webserver. The employed docking protocol was similar to the one discussed above except that we have increased exhaustiveness of search to a maximum value of eight. For this analysis, we used AutoDockvinaversion 1.1.2 installed on our local machine [20]. UCSF chimera version 1.13.1 was used to prepare both ligands and target, it allows user to add both polar hydrogen atoms and Gasteiger charges and it also ignores water molecules and non-standard residues.Additionally it provides a user friendly graphical interface to access AutoDockvina. We used MarvinSketch version 19.19[21] to draw both 2d and 3d chemical structures of the selected hits.The generated ligand-protein complex was saved as PDB file for the least binding energy pose. It was then visualized by using discovery studio Visualizer version 19.1.0 [22] in order to analyze both docking conformation and types of bonding involved.

### Pharmacokinetics and toxicology prediction:

We used two webservers, namely pkCSM [23] and Swiss ADME[24], to predict different pharmacokinetics features and drug-likeness model scores for the top five hits. These online platforms implement both molecular similarity and predictive regression to analyze submitted molecules[25,26]. We were also able to estimate median lethal dose (LD50) for these compounds by using ProTox-II server[27,28].

## Results

**Figure 3:**
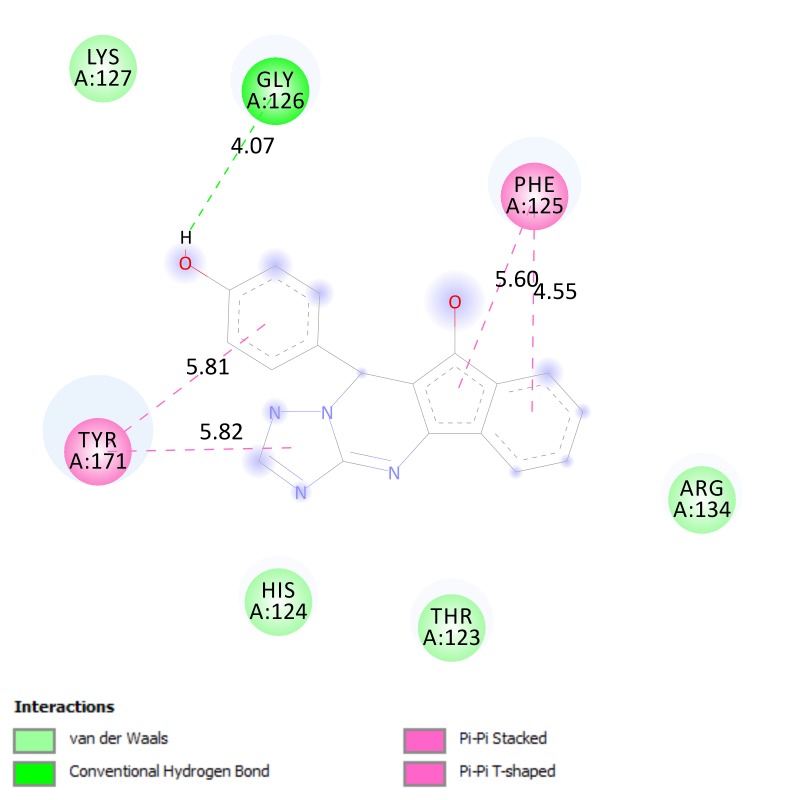
Two dimensional illustration for interaction between compound 5 and matrix protein VP40. Amino acid residues are represented as colored discs while bonds are sketched as dashed lines with predicted bonds length number.

The chemical structures and characteristics for the top five hits as screened virtually against Ebola virus VP40 protein are shown inFigure 2 andTable 1 respectively. The compounds were ordered according to their minimum binding energy to VP40.The minimum binding energy, pharmacokinetics and toxicology features are estimated for these selected compounds as seen in Table 2. According to this table, the average binding energy for these chemicals was between -7.0 and -5.9 kcal/mol. These compounds follows Lipinski's rule of five except compound 2 and 3. Only compound 5 shows high drug-likeness score and may be a potential lead candidate. All these selected hits have high percentage of predicted intestinal absorption with relativelylow volume of distribution. Total clearance ranges from 1.3 to 3.6 ml/min/kg for these five compounds. Unfortunately, compound 2 had failed to pass virtual AMES test and it may possess a mutagenic potential. Both compounds 2 and 5 have relatively high median lethal dose (LD50) as compared to the others.Docking image as seen in Figure 3 had revealed that compound 5, our probable lead candidate, is clearly involved in conventional hydrogen bond with Glycine 126 residue. It is also engaged in Pi hydrophobic interaction and van der Waals (vdW) bonding with Phenylalanine 125 and Arginine 134 respectively.

## Discussion

Due to its high fatality rate and transmission capacity, Ebola virus has been recognized as type "A" bio-weapon microorganism [1,2]. Management of Ebola hemorrhagic fever was focused towards symptoms mitigation and related complications control. Recently, a potential Ebola vaccine and a monoclonal antibody medication have been developed [3,4,29].Furthermore, target based virtual high throughput screening approach has been implemented to identify potential anti-EboV hits[2]. In this trend, Tamilvanan and Hopper virtually screened both Traditional Chinese medicine (TCM) database and Asinex database against VP40 crystal with code 1H2C. By using Glide based three-tiered docking strategy, they were able to report five natural and five synthetic potential inhibitors of Ebola virus VP40. Of these ten possible inhibitors, compound ASN03576800 (2-[2(1,3-benzodioxol-5-ylamino)-2-oxoethyl] sulfinylacetic acid) had displayed a promising minimum binding energy and an interesting orientation within VP40 active site pocket. Interestingly, these two researchers had used a docking grid box coordinates very close to those used in our project [16].Additionally, Abazari et al. 2015 had screened 120,000 compounds from zinc database for potential inhibitors of matrix protein VP40 (code 4LDB). By using Autodockvina for virtual screening[20], they were able to report four drug-like chemicals with binding energy range from -11.3 to 10.1 kcal/mol[15].Later on, Alam El-Din et al. 2016 had searched PubChem database for conformers of pyrimidine, 2,4 dione by using similarity fingerprints. The retrieved 1800 compounds were virtually screenedagainst VP40 crystal of Sudan Ebola virus by using AutoDock 4 [30]. Then, they had employed virtual ADMET web tools to estimate pharmacokinetics and toxicity properties for these compounds. They were able to report seven hits with promising minimum binding energy and low virtual toxicity [29].Recently, Nagarajan et al. 2019 had virtually evaluated 48 sugar alcohols for binding to 1H2C crystal of VP40. By using both virtual docking and molecular dynamics (MD) simulations, they were able to find that Sorbitol had the best binding affinity to VP40 crystal. Sorbitol-VP40 complex was also stable throughout MD simulation period [31]. In our project, we have applied structure based virtual screening to recognize novel compounds with potential capacity to interfere with Ebola virus VP40 function. We have used mcule.com platform with different filters to accelerate the screening of more than 42 million compounds database. Various pharmacokinetics and toxicology characteristics have been estimated by using prediction regression webservers.

According to Table 2, all the screened compounds have good virtual binding potential to matrix protein VP40. These five hits have high predicted percentage of intestinal absorption and low volume of distribution. Only compound 5 represents a potential lead candidate as it has high drug-likeness model score and lead-likeness characteristics. A compound may exhibit lead-likeness behavior if it has the following three features [32]: (1) The molecular weight should be between 250 g/ mol and 350 g/ mol; (2) The partition coefficient (XLOGP3) should be ≤ 3.5; (3) The number of rotatable bonds should be ≤ 7.Additionally, compound 5 has the highest estimated water solubility among the five hits. This compound has high predicted median lethal dose (LD50) and no mutagenic potential and may be relatively safe compound.Two dimensional docking as shown in Figure 3 shows that compound 5 is well involved in hydrogen bonding with Glycine 126 amino acid residue. It is also involved in multiple hydrophobic and Van der Waals interactions with Phenylalanine 125, Arginine 134 and Tyrosine 171 residues. This virtual behavior may enable compound 5 to effectively interfere with VP40-RNA interaction and VP40 octamer formation. Further in vitro analysis may be required to evaluate the impact of compound 5 on Ebola virus replication.

## Conclusion

The known protein target of VP40 from Ebola virus was screened with 42 million compounds in mcule.com database for potential lead inhibitors.The filtered hits were ranked based on their minimum binding energy and the top five compounds were selected for detailed study. Various pharmacokinetics and toxicology properties were estimated by using available predictive regression and molecular similarity tools. The lead compound #5 ((10R)-10-(4-hydroxyphenyl)-11,12,14,16-tetraazatetracyclo[7.7.0.0^2^,^7^.0^11^,^15^] hexadeca-1(16),2(7),3,5,8,12,14-heptaen-8-ol) possesses high drug-likeness score and displays lead-likeness features. Molecular docking analysis shows that compound #5 forms optimal hydrogen bonding with hydrophobic interactions at the active site of the matrix protein VP40. The compound also has high LD50 value as compared to other filtered hits. Therefore, the lead compound #5 is recommended for further consideration and evaluation using in vitro and in vivo models

## Figures and Tables

**Table 1:** Chemical properties of the top five compounds virtually screened against VP40 of Ebola virus. Compounds are ordered according to their minimum binding energy to VP40 of Ebola virus

Compound No	Molecular Formula	M.W. (g/ mol)	Log P	PSA (Å^2^)	H-bond acceptors	H-bond donors
1	C23H25N3O3S	423.529	4.853	96.01	6	2
2	C23H23N3O	357.447	5.988	44.7	4	1
3	C22H23FN2O3S	414.494	5.305	66.07	5	0
4	C24H21N5O3	427.454	3.981	85.76	8	0
5	C18H12N4O2	316.313	2.4258	83.53	6	2

**Table 2:** Predicted minimum binding capacity, pharmacokinetics features and toxicity profile for the top five compounds screened against Ebola virus VP40. Compounds are arranged according to their minimum binding energy to VP40 of EboV

Property	Compound #1	Compound #2	Compound #3	Compound #4	Compound #5
Average binding energy (Kcal/ mol)	-7	-6.8	-6.5	-6.4	-5.9
Rule of five (RO5) violations	0	1	1	0	0
Drug-likeness model score	-0.09	0.03	0.15	0.42	1.12
Lead-likeness	No	No	No	No	Yes
Water solubility (mg/ Kg)	1.12e-02 (Moderate)	2.11e-03 (Moderate)	4.02e-03 (Moderate)	9.69e-03 (Moderate)	5.59e-02 (Soluble)
Percentage of intestinal absorption (human)	89.6	87.7	95.8	100	94.9
Steady state volume of distribution (VDss) (L/Kg)	3.5	3.6	0.6	1.5	2.2
Total clearance (ml/min/kg)	1.7	2.2	1.5	3.6	1.3
AMES test (mutagenicity)	No	Yes	No	No	No
Predicted median lethal dose (LD50) (mg/Kg)	600	2120	575	300	2025

**Figure 2:**
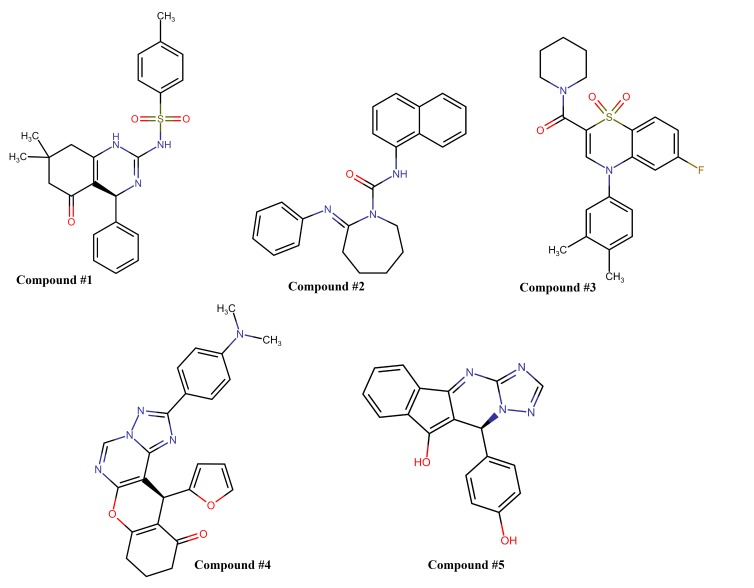
Chemical structures for the top five hits screened virtually against Ebola virus VP40. The compounds were ordered according to their minimum binding energy.
